# Food Noise: Emerging Concept, Neurobiological Mechanisms, and Psychopathological Implications

**DOI:** 10.1192/j.eurpsy.2026.10726

**Published:** 2026-07-31

**Authors:** M. F. Fernandes, J. L. Guimarães, A. Sousa, C. N. Pereira, M. A. Mateus

**Affiliations:** Psychiatry, ULS Castelo Branco, Castelo Branco, Portugal

## Abstract

**Introduction:**

“Food noise” is an emerging term, originated from patient’s anecdotes, that inspired a wider conversation in the media and amongst clinicians. Since it is not a formal diagnosis, its definition varies throughout the available literature, one of them being *“persistent thoughts about food that are perceived by the individual as being unwanted and/or dysphoric and may cause harm to the individual, including social, mental or physical problems”.* It has become more noticeable through its sudden absence, reportedly following treatment with glucagon-like peptide-1 receptor agonists (GLP1-RAs). Understanding its neurobiological and psychopathological underpinnings may provide valuable insights for psychiatry and metabolic medicine.

**Objectives:**

This review aimed to synthesize current evidence on food noise, focusing on its conceptual definition, possible physiological and psychopathological mechanisms, and implications for clinical practice in psychiatry.

**Methods:**

Original scientific articles and other relevant scientific publications indexed in the databases - *PubMed, Google Scholar, ScienceDirect,* and *APA PsycNet* – were analyzed. Preference was given to studies published in the last 10 years, written in English. Articles were examined for conceptual frameworks, neurobiological findings, and clinical implications related to food noise.

**Results:**

Food noise has been conceptualized as heightened reactivity to food cues, reflecting persistent activation of reward-related brain circuits (insula, amygdala, parietal cortex, hypothalamus). This state of reactivity can lead to food-related intrusive thoughts and negatively impact daily life, mirroring the preoccupation and disordered thinking observed in behavioral addictions and substance use disorders.

Functional MRI studies demonstrate that GLP-1 receptor agonists, such as liraglutide, modulate brain responses to highly palatable food cues, supporting a physiological basis for the reduction in food noise reported by patients. Preclinical and translational evidence suggests GLP-1 signaling influences dopaminergic and reward pathways, potentially attenuating compulsive behaviors beyond eating. Psychopathologically, food noise aligns with intrusive thought processes observed in eating disorders and obsessive-compulsive spectrum disorders.

**Conclusions:**

Food noise is a psychological construct bridging psychiatry, neuroscience, and metabolic medicine. Although still colloquial, it may serve as a useful framework to understand intrusive food-related cognitions and their modulation by neurobiological pathways, particularly GLP-1 signaling. The role of food noise in weight management and mental health, as well as its social and cultural implications requires further research in order to validate operational definitions, develop standardized measures, and explore implications for diagnosis and treatment within psychiatric populations.

**Disclosure of Interest:**

None Declared

